# Cases of Premature Delivery

**Published:** 1845-04

**Authors:** 


*Cases of Premature Delivery.* By M. SENLEN.—M. Senlen has excited labour prematurely 13 times in 7 women, in consequence of deformed pelvis. In 3 of these the deformity was produced by rickets; in 4 by malacosteon. Of the 13 labours in these 7 women only 7 children were born alive, and 4 of these died very shortly after birth. The antero-posterior diameter of the pelvis in these women was 2 inches in one, 2½ in another, 2¾ in another, and in the rest less than 3 inches. Labour was induced by puncturing the membranes, and according to the contraction of the pelvis, was brought on from the 29th to the 38th week of pregnancy, The average period, however, was on the completion of the 30th week, or 7 calender months. —*Ibid, from L'Experience.*

